# A Rare Manifestation of Systemic Lupus Erythematosus: A Case of Lupus Cystitis in Puerto Rico

**DOI:** 10.7759/cureus.79741

**Published:** 2025-02-27

**Authors:** Luis Xavier Mayol-Velez, Saribel Torres, Nayaret Rivera, Hassan Issa, Fernando Zayas

**Affiliations:** 1 Family Medicine, University of Puerto Rico, Medical Sciences Campus, San Juan, PRI

**Keywords:** academic hospitalist, academic rheumatology, lower urinary tract symptoms, lupus, lupus cystitis, urinary

## Abstract

Lupus cystitis is an uncommon, yet serious complication of systemic lupus erythematosus (SLE) marked by inflammation of the bladder and associated with significant urinary tract manifestations. The diagnosis of lupus cystitis can be very challenging as it often appears before the diagnosis of lupus has been established. This report presents the case of a 36-year-old male patient with a history of SLE admitted with gastrointestinal (GI) and urinary symptoms who was subsequently diagnosed with lupus cystitis and highlights the clinical presentation, diagnostic challenges, and management strategies in these patients. The diagnosis was confirmed through a combination of clinical examination and laboratory findings. Treatment with corticosteroids and immunosuppressive agents can lead to substantial improvement for which early diagnosis and recognition by primary care team providers in a hospital setting is pivotal as delayed diagnosis can result in significant morbidity. This report highlights the critical importance of considering lupus cystitis as a differential diagnosis in patients with systemic SLE patients presenting with urinary symptoms.

## Introduction

Systemic lupus erythematosus (SLE) is an autoimmune disease that can affect multiple organs, including the kidneys, skin, joints, and nervous system, and it has been reported it also affects the urinary tract. The pathophysiology of SLE is mainly associated with the production of specific antibodies against DNA and histones. Immunological alterations, particularly the production of various antinuclear antibodies (ANA), are a determining feature of the disease [1]. The majority of patients with lupus present with a constellation of non-specific symptoms including fatigue, fever, rash, and arthritis, and almost three-fourths of patients have renal manifestations at initial presentation [2]. Lupus cystitis, although rare, is a recognized manifestation of SLE that can cause significant morbidity. It is characterized by symptoms such as urinary frequency, urgency, dysuria, and suprapubic pain, which can often be misdiagnosed as a urinary tract infection (UTI) in some patients [3]. The interval from onset of SLE and further development of hydroureteronephrosis and further development of lupus cystitis varies from zero to five years. Moreover, although this disease is genitourinary, the most common clinical manifestations are gastrointestinal (GI) symptoms, while urinary symptoms are less common and relatively mild [4]. 

Lupus cystitis is a rare condition, affecting 0.5-2% of SLE patients. It shows geographic variation, with higher prevalence reported in Asian populations, particularly in studies from Japan and China. Specific data on the prevalence of lupus cystitis in Puerto Rico is scarce. However, Puerto Rico has one of the highest global rates of SLE, the underlying condition linked to lupus cystitis. The prevalence of SLE in Puerto Rico has not been established but a limited study of submitted claims found an overall prevalence of 159 per 100,000 individuals [5], suggesting a significant presence of lupus-related complications. Lupus cystitis commonly presents with both urinary and GI symptoms. The most frequent urinary symptoms include urinary frequency, urgency, and dysuria. However, these symptoms are often mild and less prominent compared to GI manifestations. GI symptoms such as nausea, anorexia, vomiting, abdominal pain, and diarrhea are typically more severe and can overshadow the urinary complaints as seen with this patient. 

Although lupus cystitis is a rare complication of SLE, it may lead to permanent bladder dysfunction, and its complications may include irreversible impairment of renal function [6]. The pathogenesis encompasses immune complex deposition and subsequent inflammation of the bladder wall which leads to dysfunction [3,7]. Early diagnosis and appropriate treatment are essential to prevent complications such as bladder fibrosis and reduced bladder capacity which may cause permanent damage.

## Case presentation

A 36-year-old male patient with a past medical history of SLE presented to the emergency department with complaints of intractable vomiting and diarrhea that started two weeks before his arrival. He reported that symptoms started after a day of binge drinking where he consumed more than five drinks of hard liquor. As per the patient, his symptoms were getting worse each day without any improvement. In fact, for the last eight months, the patient reported having an increase in urinary frequency, urgency, and suprapubic pain. Given this presentation, the patient was admitted to our institution. During the first day of the hospitalization, the patient reported worsening of his urinary symptoms with increased urinary effort and low urinary output. Prior to this hospitalization, his SLE had been managed with hydroxychloroquine 200 mg POD and Prednisone 20 mg BID. 

Upon examination, the patient had stable vital signs. Physical examination was grossly unremarkable except for suprapubic tenderness on abdominal palpation. Laboratory tests revealed elevated serum creatinine, proteinuria, and hematuria. Urinalysis showed pyuria, but urine cultures were negative for bacterial growth. Anti-double-stranded DNA (anti-dsDNA) antibody titers were elevated and complement levels (C3, C4) were both decreased (Table 1). 

**Table 1:** Remarkable laboratory values

Laboratory	Results	Reference Range
Serum Creatinine	1.49 mg/dL	0.52 - 1.04 mg/dL
Urine Protein	>1000 g/day	Negative
RBC	> 10 RBC/HPF	0-5 RBC/HPF
Anti-Double-Stranded DNA titer	13 IU/mL	< 4.0 IU/mL - Negative, 5.0 - 9.0 IU/mL - Indeterminate, > 10.0 IU/mL: Positive
C3	60 mg/dL	87 - 100 mg/dL
C4	10 mg/dL	19 - 52 mg/dL

**Figure 1:**
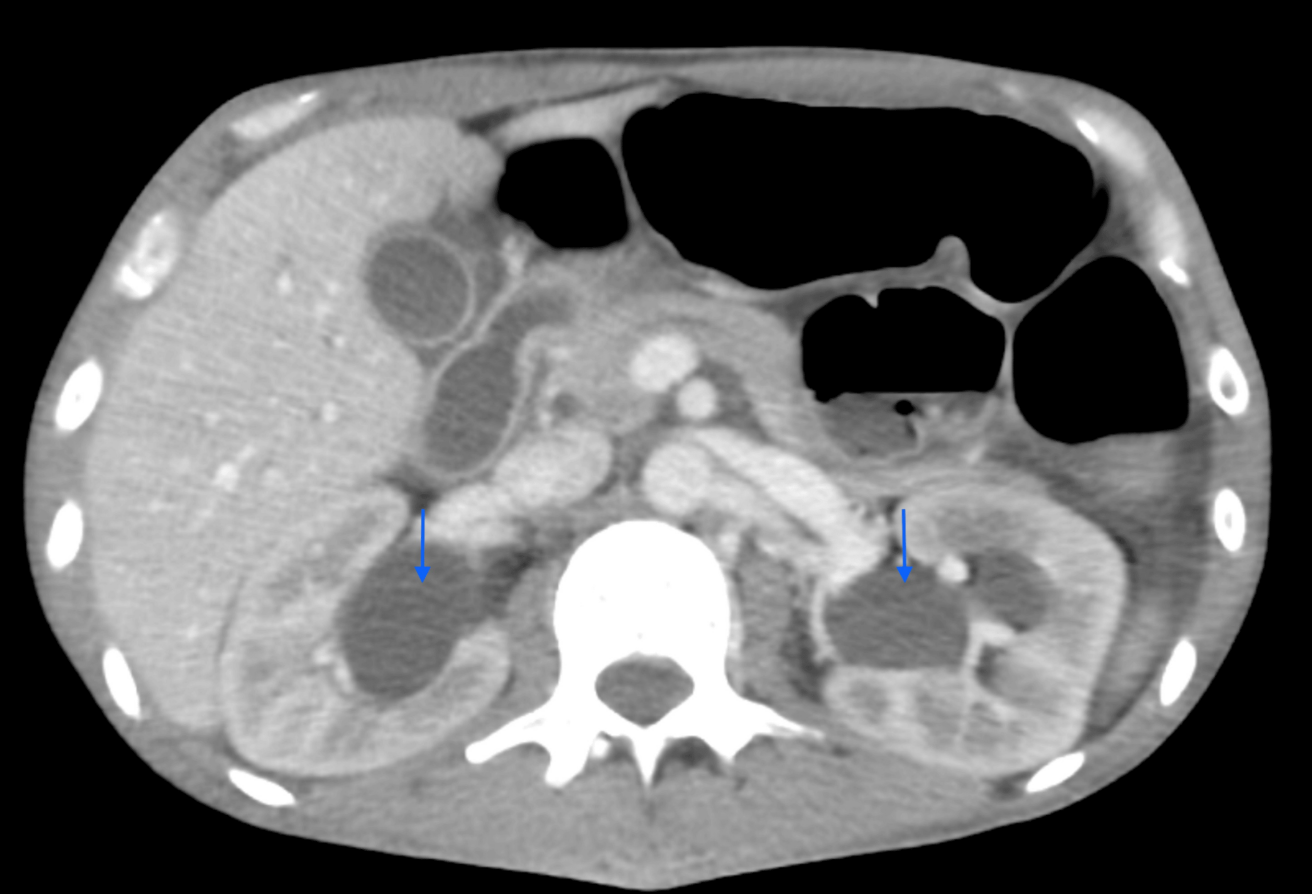
CT of abdomen and pelvis showing bilateral hydroureteronephrosis

Imaging was ordered for further assessment. Abdominal CT w/o contrast demonstrated bilateral hydroureteronephrosis and thickened urinary bladder with no renal calculi or signs of obstruction (Figure 1). Rheumatology and Urology services were consulted. A bedside ultrasound of the abdomen revealed a thickened bladder wall with reduced capacity. The patient was admitted for the management of lupus flare-up with GI organs as a primary target. 

During the hospital course, urinary symptoms kept worsening despite improvement in nausea, vomiting, and diarrhea. Six days after admission, the patient developed marked suprapubic distention and tenderness, with no urinary output. After proper foley placement, 2100 mL of urine output was obtained. At this point, consultants recommended a biopsy of the bladder mucosa. A biopsy was performed and showed benign urothelial mucosa exhibiting mild reactive changes, negative for evidence of dysplasia and negative for evidence of overt malignancy. 

As per clinical findings and ordered workup, obstructive and infectious etiologies were ruled out for which the diagnosis of lupus cystitis was concluded. This patient was treated with high-dose steroids consisting of methylprednisolone 1,000 mg IV for three days. The patient was further transitioned to cyclophosphamide infusion, as per Rheumatology services recommendations. The patient was also started on mesna in order to prevent hemorrhagic cystitis. Medications were tolerated adequately with subsequent improvement in urinary symptoms. The patient was able to be discharged home to continue with rheumatology and urology follow-up.

## Discussion

Lupus cystitis is a rare, uncommon but severe complication of SLE, with an estimated prevalence of 0.5-1.0% in SLE patients [7]. Although the exact pathophysiology remains unclear, the current data suggest that immune complex deposition and complement activation are believed to play a central role in bladder inflammation and further development of the disease [8]. The clinical presentation may be similar to urinary tract infections [1].

Imaging studies, such as ultrasound and cystoscopy, are crucial for an accurate diagnosis. Moreover, ultrasound findings typically include a thickened bladder wall and reduced bladder capacity, while cystoscopy may reveal a trabeculated bladder with inflamed mucosa for which urology services should be involved in the case [9]. In addition, histological examination confirms the diagnosis by showing chronic inflammatory infiltrates in the bladder wall [10].

The management of lupus cystitis involves aggressive immunosuppressive therapy to control inflammation and prevent further bladder damage. Corticosteroids are the mainstay of treatment, often combined with immunosuppressive agents such as cyclophosphamide or mycophenolate mofetil [11]. In this case, the patient was started on high-dose prednisone and cyclophosphamide. Over the course of two weeks, his urinary symptoms significantly improved, with a marked reduction in urinary frequency and suprapubic pain. 

## Conclusions

Lupus cystitis, though rare, is a significant manifestation of SLE that requires prompt recognition and treatment. This case highlights the importance of considering lupus cystitis in SLE patients presenting with urinary symptoms, particularly when typical UTIs have been ruled out. Early diagnosis through imaging and cystoscopic evaluation, coupled with aggressive immunosuppressive therapy, is essential for optimal patient outcomes. Further research is needed to better understand the pathogenesis and develop targeted therapies for lupus cystitis. This case report highlights that a thorough clinical evaluation can effectively diagnose lupus cystitis, reducing the need for invasive biopsies and reducing comorbidities in this population. 
